# Genome Sequence of *Arthrobacter siccitolerans* 4J27, a Xeroprotectant-Producing Desiccation-Tolerant Microorganism

**DOI:** 10.1128/genomeA.00526-14

**Published:** 2014-06-19

**Authors:** M. Manzanera, L. Santa-Cruz-Calvo, J. I. Vílchez, C. García-Fontana, G. A. Silva-Castro, C. Calvo, J. González-López

**Affiliations:** Institute for Water Research and Department of Microbiology, University of Granada, Granada, Spain

## Abstract

We report the first genome sequence for *Arthrobacter siccitolerans* 4J27, a newly described desiccation-tolerant species. The complete genome of *A. siccitolerans* 4J27 has been sequenced and is estimated to be around 5.3 Mb in size, with an average GC content of 65.13%. We predict 4,480 protein-coding sequences (CDSs).

## GENOME ANNOUNCEMENT

The genus *Arthrobacter* belongs to the class of high-GC-content *Actinobacteria*. Species of *Arthrobacter* have been found in common soils and in extreme environments, including dry soils (1). *Arthrobacter siccitolerans* 4J27 is a highly desiccation-tolerant new species (2, 3). Many desiccation-tolerant microorganisms produce xeroprotectants to protect themselves against damage caused by drought (4–10) and other stressors (11). Thus, the synthesis of nonreducing disaccharides such as trehalose and sucrose is associated with the ability of these organisms to survive in the dry state (5, 9–11). We have reported the production of trehalose, glucose, and glutamine at relative concentrations of 5.8:2.8:1 as a xeroprotectant designated S4J27-D (2). To our knowledge, the complete genome sequence of *A. siccitolerans* had not yet been deposited in the DDBJ/EMBL/GenBank databases. In this study, we determined the whole-genome sequence of *A. siccitolerans* 4J27 with pyrosequencing technology as implemented by the 454 Life Science–Roche platform (12). Sequencing was provided by Lifesequencing S.L. (Valencia, Spain) with a combined shotgun and 8-kb mate-pair sequencing approach.

A total of 142,567 reads were produced, with an average read length of 623 bases and 90,146 sequences for the shotgun approach and an average read length of 412.81 bases for the mate-pair sequencing strategy. The total number of sequenced bases is 88,868,818, representing a sequencing depth of around 23×. *De novo* assembly was performed with default parameters using Newbler assembler v. 2.6. The assembly resulted in 64 contigs, 50 of which were larger than 500 bp. The *N*_50_ of the contig assembly was 212,564 bp, and the largest contig was 628,521 bp. Most of these contigs were ordered in three scaffolds (based on mate-pair information), where the largest scaffold was 4,789,105 bp. This combination of scaffolds and contigs resulted in an estimated genome size of 5.3 Mb. Gap closure was attempted with gap-spanning clones and PCR products. Putative coding sequences were predicted and genes were annotated with a pipeline implemented at Lifesequencing S.L. Briefly, protein-coding sequences (CDSs) were predicted by the combined use of Glimmer (13–15), RNAmmer (16), tRNAScan (17, 18), and BLAST (19, 20). The complete genomic information for *A. siccitolerans* 4J27 is contained on three scaffolds, one of which is apparently the circular 4,789,105-bp chromosome with an average GC content of 65.15%. The genome was found to contain 4,536 protein-coding genes, 2 rRNA operons, and 51 tRNA genes.

Analysis of this genome sequence data leads us to propose the presence of all three known pathways for trehalose biosynthesis in the main component of the xeroprotectant mixture, i.e., from UDP-glucose and glucose 6-phosphate (OtsA-OtsB pathway), from malto-oligosaccharides or α-1,4-glucans (TreY-TreZ pathway), or from maltose (TreS pathway). This knowledge can lead to advances in biotechnological applications for anhydrobiotic engineering (5, 8, 11).

The complete genome sequence of *A. siccitolerans* 4J27 will contribute to the development of xeroprotectant mixtures with biotechnological applications.

### Nucleotide sequence accession numbers.

The complete genome sequence of *A. siccitolerans* 4J27 has been deposited in the DDBJ/EMBL/GenBank databases under accession numbers CAQI01000001 to CAQI01000064.
